# A High-Throughput Assay for Congenital and Age-Related Eye Diseases in Zebrafish

**DOI:** 10.3390/biomedicines7020028

**Published:** 2019-04-11

**Authors:** Lindy K. Brastrom, C. Anthony Scott, Deborah V. Dawson, Diane C. Slusarski

**Affiliations:** 1 Department of Biology, University of Iowa, Iowa City, IA 52245, USA; melinda-brastrom@uiowa.edu; 2 Department of Pediatrics, Baylor College of Medicine, Houston, TX 77030, USA; Charles.scott@bcm.edu; 3 Departments of Biostatistics and Pediatric Dentistry, University of Iowa, Iowa City, IA 52245, USA; deborah-dawson@uiowa.edu

**Keywords:** vision, visual assay, optomotor response, visual impairment disorders, microphthalmia, cataracts, *rbm24a*, *crim1*, zebrafish

## Abstract

Debilitating visual impairment caused by cataracts or microphthalmia is estimated to affect roughly 20 million people in the United States alone. According to the National Eye Institute, by 2050 that number is expected to more than double to roughly 50 million. The identification of candidate disease-causing alleles for cataracts and microphthalmia has been accelerated with advanced sequencing technologies creating a need for verification of the pathophysiology of these genes. Zebrafish pose many advantages as a high-throughput model for human eye disease. By 5 days post-fertilization, zebrafish have quantifiable behavioral responses to visual stimuli. Their small size, many progeny, and external fertilization allows for rapid screening for vision defects. We have adapted the OptoMotor Response to assay visual impairment in zebrafish models of cataracts and microphthalmia. This research demonstrates an inexpensive, high-throughput method for analyzing candidate genes involved in visual impairment.

## 1. Introduction

The World Health Organization estimates visual impairment caused by either congenital or age-related factors affects 1.3 billion people worldwide, making visual impairment and blindness a major public health concern. Our understanding of the genetic contribution to visual disorders has increased as a result of improved genomic technologies. The accelerated rate of discovery of candidate genes associated with visual disorders creates the need for rapid verification of the pathophysiology of these candidate genes. While the mouse has served as a model for understanding visual impairment genes, mice are rod-dominant and not well suited for high-throughput visual analysis [1,2,3,4]. The zebrafish show rapid development with an eye rudiment present by 24 h and the ability to respond to light on the third day of development. This rapid development, coupled with large clutch sizes and rod/cone utilization similar to that of humans, makes zebrafish amenable to high-throughput visual screens [5,6].

Our lab has previously developed a high-throughput approach assay to detect loss of visual function in zebrafish which we have named VIZN (Visual Interrogation of Zebrafish maNipulations) [7]. In this assay, we use an automated tracking device and we developed software for the analysis of the vision startle response, which tests the ability of zebrafish larvae to detect changes in light. The vision startle response works by placing a larval zebrafish in each well of a multi-well plate. The motion of larvae is tracked over a roughly 33 min time period. The first 30 min track the larvae’s motion in a constant light environment to determine baseline activity. Then, five regularly-spaced interruptions of the light follow. The vision startle response assay uses interruptions in light to produce a shadow passing over the larvae. The larvae see this shadow and interpret it as a predator. To avoid predation, they abruptly swim in a different direction. The data from the motion tracking device is uploaded to the VIZN program for analysis of the frequency of larval responses to the light change.

While the vision startle response is a reliable assay for blindness, animals with reduced visual function can still maintain light perception and accordingly, may show a similar VIZN response as wild-type larvae. We set out to develop an assay to investigate varying degrees of visual impairment. There are currently two assays that analyze visual impairment: the optokinetic response (OKR) and the optomotor response (OMR). The OKR and the OMR utilize similar technology as both use an animation of a series of black and white lines to simulate motion and produce a response from the subject [8,9,10,11]. The OKR involves a revolving drum where a single zebrafish is immobilized and placed in the middle. When the drum is rotated, the zebrafish sees the lines, interprets them as motion, and attempts to swim in the direction of the rotation. Because the zebrafish has been immobilized, only its eyes are able to move. Thus, the response of the zebrafish is determined by its eye movements while it tracks the revolving pattern [12]. The disadvantage of the OKR for a high-throughput approach is that it screens a single subject at a time and requires video analysis to evaluate eye movements [13,14,15].

In contrast, the OMR can be adapted to screen multiple larvae at a time. Instead of using a moving drum, the OMR projects the black and white pattern underneath a plate of zebrafish larvae [16]. The black and white lines mimic sinusoidal waves larvae would encounter in a stream [15]. Visually responsive larvae see the animation and then process the information to interpret the lines as motion. When larvae are exposed to the animation, they will change their direction of movement to align with the perceived motion. Their response to the motion is rapid with zebrafish larvae aligning within 30 s. The extent of alignment, or response to the animation, can be scored. Larvae can be placed in multi-well plates which allows many animals to be screened at a time. This coupled with the rapid scoring process make the OMR an ideal assay to screen for visual impairment in a high-throughput manner.

Congenital and age-related factors contribute to visual impairment. Microphthalmia is a congenital condition caused by either genetic or environmental factors in which one or both eyes are abnormally small [17]. The incidence of microphthalmia is estimated to fall between 3 and 30 per 100,000 people [18]. While some patients with microphthalmia are visually responsive, a majority are not, which makes microphthalmia a life-long problem to their vision health [17].

One gene associated with microphthalmia is *rbm24a* [19]. The RNA binding motif protein 24a (Rbm24a) is a protein involved in alternative splicing and the stabilization or degradation of RNA [20,21,22,23]. The RNA regulatory activity of the Rbm24a protein is due to the RNA recognition motif domain [24]. The zebrafish Rbm24a protein shows high similarity with the mouse (90.3%) and human (91.1%) proteins. Prior studies showed strong expression in the lens, heart, and somites of zebrafish, *Xenopus*, and mouse [25,26,27,28,29]. Using morpholino (MO) knockdown in zebrafish, a previous study showed a microphthalmic phenotype [25]. At higher doses, a microphthalmic phenotype is observed along with cardiac and somite defects [30]. Cardiac defects cause premature death in zebrafish around 10 days post-fertilization (dpf). A similar phenotype is shown in mice. *Rbm24* mutant mice display severe cardiac defects which leads to death around embryonic day 13.5 making visual studies in the mouse model difficult [20].

In addition to microphthalmia, we also wanted to examine cataracts. Cataracts are broadly defined as any opacity of the lens and are the main cause of blindness worldwide [31,32,33]. Cataracts can be a congenital or an age-related condition. Between 8.3–25% of congenital cataracts are hereditary with the remainder caused by prenatal factors such as an intrauterine infection [34,35,36]. Age-related cataracts are caused by many factors including UV exposure, smoking cigarettes, and steroid use [31]. Both congenital and age-related cataracts can cause permanent damage to vision and even blindness if not treated promptly.

A gene that has been implicated in cataracts in mice is *Crim1* [37]. Cysteine-rich motor neuron 1 (*Crim1*) encodes a protein which contains an extracellular N-terminal insulin-like growth factor-binding protein motif, two Von Willebrand factor type C domains, four antistasin-like domains, four more Von Willebrand factor type C domains, followed by a transmembrane domain and a cytoplasmic C-terminal. A three-generation family with parts of three exons deleted display colobomatous microphthalmia and microcornea (OMIM entry 606189) [38,39]. In hypomorphic and null mutant mouse models, *Crim1* has been shown to cause congenital cataracts, aphakia, and additional organ defects in the ears, urogenital tract, and kidneys [37,40]. These defects are attributed to CRIM1 interacting with growth factors including TGFβs, BMPs, and VEGFs [41]. The presence of these additional organ defects results in perinatal lethality in *Crim1* mouse mutants which hampers analysis of their visual function [37].

Here, we describe the optimization of the OMR assay for high-throughput visual studies. We validate the approach and scoring metric by utilizing a blind genetic model. We next utilize the OMR to score two candidate genes associated with visual disorders: *rbm24a* and *crim1*. Both of these genes prove lethal in a homozygous knockout context. Due to this lethality, we identify doses of anti-sense oligonucleotide morpholinos (MO) to target *rbm24a* and *crim1* that allow for viable larvae to assess visual function. In this study, we demonstrate a low-cost approach to study visual disorders.

## 2. Experimental Section

### 2.1. Materials

The Zebrabox (ViewPoint) is used. VIZN software v1.2 analysis is performed on the data using previously described methods [7]. The OMR uses a tablet to play the OMR animation. A video camera mounted on an O-ring is used to record the movement of fish.

### 2.2. Animal Care

Zebrafish are maintained in standard conditions under the approval of the University of Iowa Institutional Animal Care and Use Committee (#8071513, 13 August 2018). Embryos are collected from natural spawning and raised between 28 and 30 °C. No more than 50 embryos were kept per 100 mm plate. Embryo plates are cleaned of dead daily and water changes are made as needed. 

### 2.3. Microinjection

1–2 cell stage embryos were injected with either a translation-blocking *crim1* morpholino (1.0–1.5, 2.5–3.0 nanograms), a translation-blocking *rbm24a* morpholino (1.0–1.2, 1.5–2.0 nanograms), and a standard control morpholino (1.0–1.5, 2.0–2.5 nanograms). Morpholinos were ordered from Gene Tools. *crim1* AUG MO sequence: 5′-AAGATACATCCTGGAGGAGGCCAT-3′. *rbm24a* AUG MO sequence: 5′-GCATCCTCACGAAACGCTCAAGTGC-3′. Standard Control MO 5′-CCTCTTACCTCAGTTACAATTTATA-3′. Microinjection needles were measured via capillary tube to ensure dosages fell in the aforementioned ranges. An app was used to calcuate the concentration of mopholino injected (https://play.google.com/store/apps/details?id=com.canthonyscott.microinjectioncalc&hl=en_US).

### 2.4. Automated Startle Response

The automated vision startle response, VIZN, was performed on 5 or 6 days post-fertilization (dpf) larvae as previously described [7]. Phenotypically normal larvae were first tested for the ability to swim by being prodded to ensure touch responsiveness and swimming ability before being sorted and placed in 48-well plates.

### 2.5. OptoMotor Response Android Application

Prior to testing, 6 dpf larvae are reared in a light environment for at least 2 h, then transferred to 48-well plates. The fish were subjected to the VIZN assay (Section 2.4) before OMR. Prior to OMR, the fish are moved to a low light, quiet space to limit external stimuli. Briefly, the OMR animation was created from a static image of alternating black and white bars. The transition as a 60° sine-wave was animated using Adobe Photoshop v19.0 to create the illusion of movement. This animation was then transferred to Adobe Premier v12.0 to create an animation consisting of an initial 5 s of solid white to note initial larval position, 30 s of alternating black and white bars to get a response, and a final 5 s of solid white to note final position. To allow for quick repetition and easy replication, an application was created to launch the animation at the press of a single button. This application was written in Java and compiled for Android to be loaded onto a 10-inch Android Tablet (source code available at: https://github.com/canthonyscott/OMR-Wave-Player). Videos were captured using a cell phone camera and the start and end positions of the larvae were scored by an investigator masked to the experimental conditions.

### 2.6. Statistical Analysis

VIZN assays were statistically evaluated using an ordinary one-way ANOVA. OMR were analyzed by either a Bowker’s test of symmetry or a Wilcoxon–Mann–Whitney test.

### 2.7. Whole Mount In Situ Hybridization

Uninjected control embryos were fixed at 3 dpf in 4% paraformaldehyde in 1× PBS and then hybridized as described [42]. Riboprobes were synthesized from a linear template and the appropriate RNA polymerases (Ambion Maxiscript Kit, Life Technologies, Waltham, MA, USA) and DIG labeling mix (Roche, St. Louis, MI, USA). Embryos were cryoprotected and embedded as described in 2.7. Primers for *rbm24a* riboprobe template were designed using NCBI Primer-BLAST. *rbm24a* Forward: 5′-CCAGGGGTTATGGATTTGTG-3′. *rbm24a* Reverse: 5′-TGCAGTTGTTGGGGTTGATA-3′. *crim1* primers for riboprobes were generated via [43].

### 2.8. Retinal Histology and Flourescent Microscopy

Uninjected control and injected embryos were fixed at 3 dpf for 24 h in 4% paraformaldehyde in 1× PBS before being submerged in 15 and 30% sucrose solutions and in OCT (optimal cutting temperature medium) overnight at 4 °C. Embryos were then aligned and embedded in OCT, frozen, and sectioned at −23 °C. Sections (8 µm) were mounted on glass slides and allowed to dry overnight. H&E staining or TOPRO3 nuclear staining was performed according to standard protocols. TO-PRO^TM^-3 (Molecular Probes, Eugene, OR, USA) stained embryos were imaged at 40× on a Leica SP8 confocal microscope.

## 3. Results

### 3.1. OptoMotor Assay for Visual Accuity

**Figure 1:**
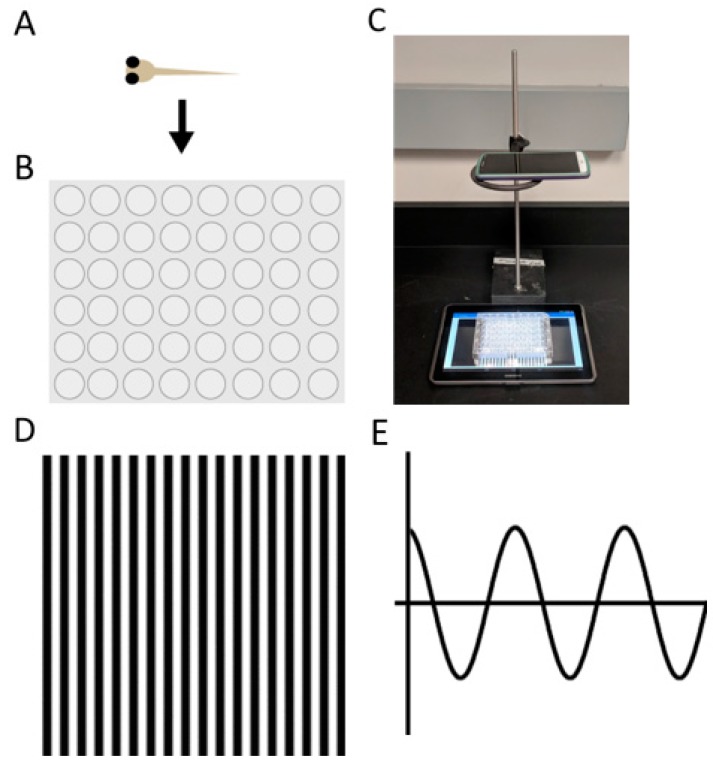
The OMR assay. (**A**) 6 days post-fertilization embryos are loaded individually into; (**B**) 48-well plates; (**C**) movement is captured with a cell phone camera on an O-ring positioned over the plate of fish on the tablet; (**D**) the tablet displays an image of black and white lines moving from right to left; (**E**) the embryos interpret the lines as sinusoidal waves and perceive this as motion.

We analyze zebrafish larvae for visual defects using a combination of the VIZN and OMR assays. VIZN uses motility tracking equipment and software (Zebrabox) [7]. OMR can be accomplished with simple equipment. Larvae are examined and sorted to only select phenotypically normal larvae into a 48-well plate (Figure 1A,B). The plate of larvae is positioned on a tablet which is positioned underneath an O-ring (Figure 1C). A cell phone or other recording device is centered on the O-ring to record the movement of larvae (Figure 1C). The tablet displays an animation of black and white lines moving from right to left (Figure 1D) [16]. The animation is patterned after a sinusoidal wave which the larvae interpret as movement and align with the pattern (Figure 1E, Supplementary Materials Movie S1).

**Figure 2:**
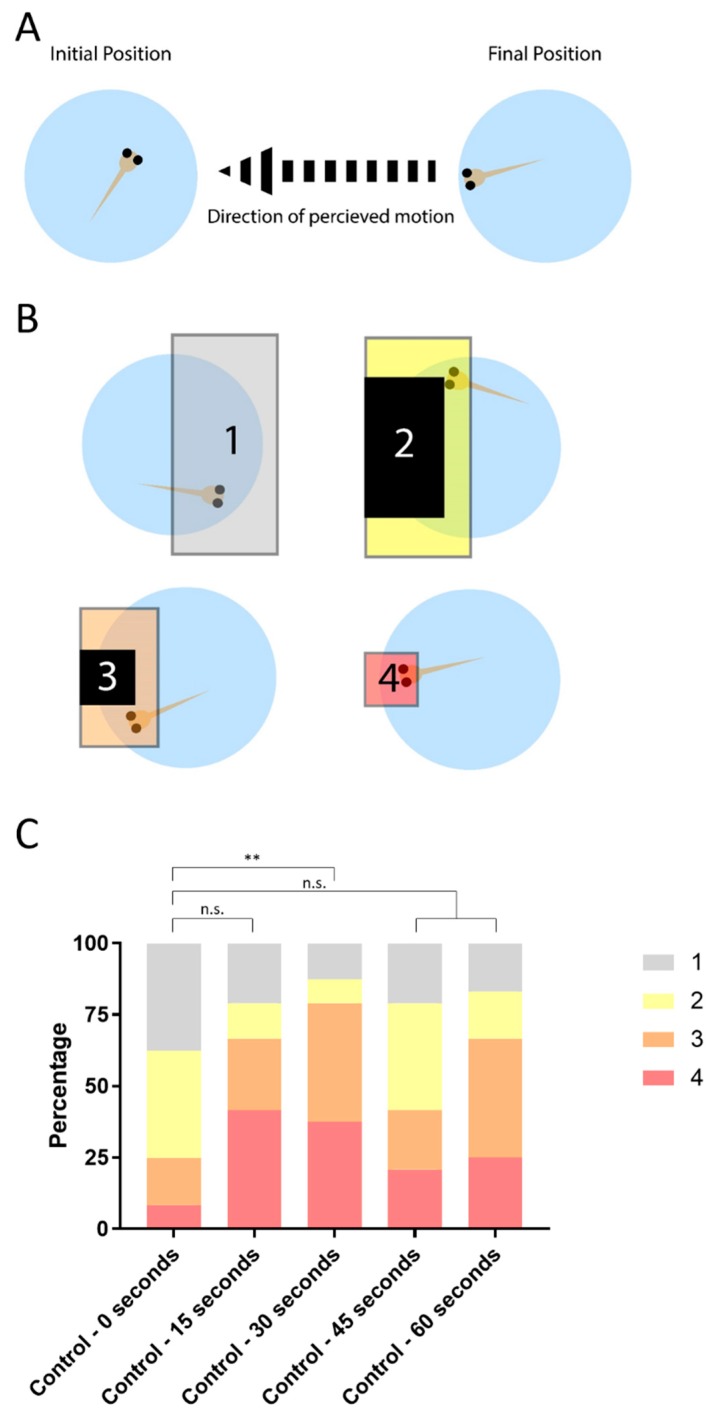
Scoring the embryo’s response to perceived motion with OMR. (**A**) Larvae start in a random orientation in the well, and respond to the OMR animation by aligning the direction of movement, to the left; (**B**) Each fish is scored based on its initial position, before the OMR, and its final position, after OMR exposure. The well is divided into four areas to indicate the level of alignment with 4 indicating high-alignment and 1 indicating a low-alignment; (**C**) 6 days post-fertilization control larvae (*n* = 24) were exposed to the OMR stimulus for a total time of 60 s and the position of the larvae scored every 15 s. Larvae with 30 s exposure show a statistically significant difference from the 0 s baseline (exact binomial test *p*-value ** = 0.0072). n.s., not significant.

We next developed a scoring system to quantify the responses to the OMR. Prior to activating the OMR animation, larvae are randomly oriented within their individual wells. After the activation of the animation, visually responsive larvae will align with the direction of the perceived motion (Figure 2A). The level of alignment is determined by the comparison of head position before the OMR animation and after the animation. We divided the well into four non-overlapping areas and labeled them as Areas 1–4 (Figure 2B). Area 1 represents low-alignment with the larva on the right 50% of the well. Areas 2 and 3 represent an intermediate-alignment as the larva is not in Area 4 but it is in the aligned-half of the well. They each account for roughly 20% of the well. Area 4 represents high-alignment with the larva in the left-most 10% of the well (Figure 2B).

We next wanted to determine the ideal exposure time to the OMR animation. In order to evaluate optimal timing, we evaluated each change in position relative to baseline at each post-0 s time point (15, 30, 45, 60 s) (Figure 2C). We classified each change in position, relative to the baseline, in terms of whether it represented improved alignment, no change in alignment, or decreased alignment. The results are shown as a bar graph representing the percentages of fish in each of the four areas over the 60 s timeframe. Before the OMR animation at 0 s, fish are randomly distributed between the four areas with roughly 50% of embryos in Area 1, 20% in each of Areas 3 and 2, and 10% in Area 4 (Figure 2C). We then assessed whether there was significant improvement in alignment relative to initial position, using two-sided exact binomial tests, at each time point. At 30 s, there was significantly more movement toward greater alignment than to decreased alignment. As seen in Figure 2C, a greater proportion of fish had moved into zones 3 or 4 at 30 s than was seen at any of the other time points considered, and it was the only time point showing significant improvement in alignment relative to baseline. Therefore, we use 30 s as the final time point for all following OMR assays and we created an application for the tablet. In this application, there is an initial 5 s of blank background (for scoring larval initial position), followed by 30 s of OMR animation and ending with a blank background (for scoring final position). The source code is available at: https://github.com/canthonyscott/OMR-Wave-Player.

### 3.2. Establishing the Masterblind Line of Fish as a Negative Control in Visual Studies

**Figure 3:**
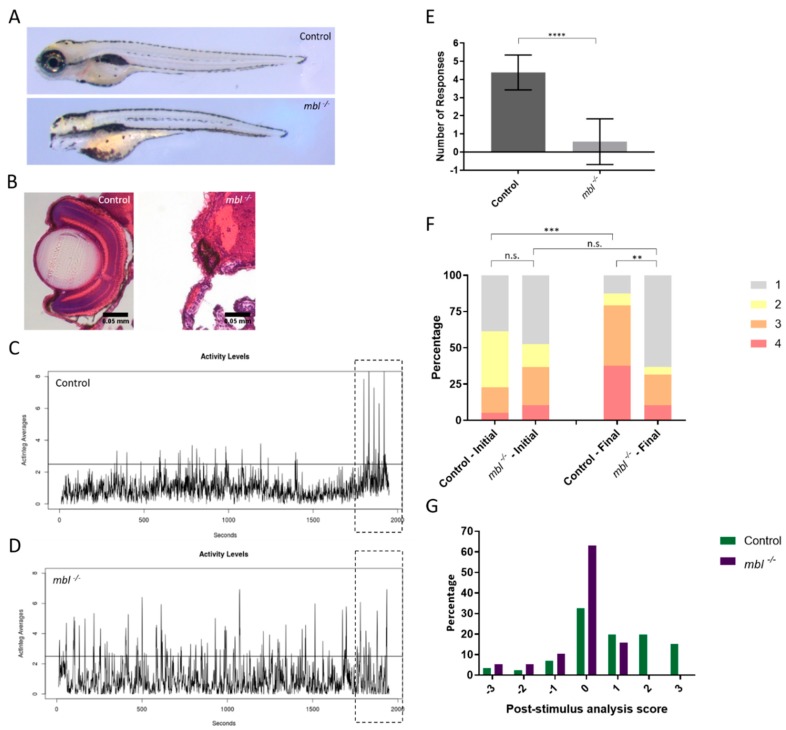
Utilizing the eyeless *masterblind* (*mbl*) mutant as a negative control for VIZN and OMR. (**A**) 6 days post-fertilization control (top) and *mbl^−/−^* mutants (bottom); (**B**) Hematoxylin and eosin staining larvae in (A). Control larvae display normal optic structures and retinal lamination while homozygous *mbl^−/−^* mutants lack eyes; (**C**) Activity profile of control and, (**D**) *mbl^−/−^*; (**E**) VIZN analysis between control (*n* = 39) and *mbl^−/−^* (*n* = 28) (Mann–Whitney, *p*-value **** < 0.0001); (**F**) OMR analysis of larvae plotted as a bar graph which shows the shifts in the population between the initial and final positions (Bowker’s test of symmetry, *p*-value *** = 0.0004; Wilcoxon–Mann–Whitney, *p*-value ** = 0.0019); (**G**) the post-stimulus analysis which takes the difference between the final and initial position to show positional changes in individual fish. The same larvae were used for all assays. Scale bars in (**B**): 0.05 mm. n.s., not significant.

We previously demonstrated the ability of the VIZN assay to evaluate candidate genes in blinding disorders [7,44,45]. We sought to establish a genetic line of zebrafish to use as a negative control. We chose the *masterblind* (*mbl*) line which has a mutation in Axin1 that abolishes its binding to Gsk3 [46,47]. Homozygous *mbl^−/−^* mutant larvae lack eyes and telencephalon (Figure 3A) [48]. Despite lacking the telencephalon which is located in the forebrain, *mbl^−/−^* larvae possess the ability to swim because the swimming circuit is located in the hindbrain [49]. Homozygous *mbl^−/−^* mutants were separated from their siblings based on morphology. Eyes were sectioned before hematoxylin and eosin (H&E) staining was performed. Uninjected control (control) zebrafish displayed normal optic structures and lamination while *mbl^−/−^* contain no optic structures nor lamination (Figure 3B).

We analyzed the visual responses of control and *mbl^−/−^* larvae. As previously described in the VIZN assay, larvae were prodded to ensure they were responsive to touch and able to swim before being placed in 48-well plates and their movement was monitored for 30 min before stimulus was applied (Movie S2–S4) [7]. Activity plots generated by VIZN established that both control and *mbl^−/−^* mutants are motile (Figure 3C,D). The dashed box to the right of each activity plot indicates the testing period, when the five interruptions of light are applied. These interruptions in light are interpreted by the fish as an approaching predator, causing them to move away from the perceived threat. The control larvae display five clear activity peaks, indicating their response to the stimulus (Figure 3C). When quantified, control larvae responded an average of 4.4 out of 5 times which indicates they are visually responsive. The *mbl^−/−^* fish fail to synchronize their total movements to the changes in light and instead display random movements for the entire duration (Figure 3D). Quantifying the *mbl^−/−^* response showed the mutant larvae responded <1 out of 5 times, indicating a lack of visual responsiveness (Figure 3E). It should be noted only *mbl^−/−^* larvae displayed this lack of response; *mbl^+/+^* and *mbl^+/−^* clutch-mates responded as well as control. 

We next evaluated both control and *mbl^−/−^* larvae by OMR. We measured the positions of fish before and after 30 s exposure to the OMR animation. We graph the results in two different ways: a population analysis and post-stimulus analysis. The population graph is a bar graph representing the percentages of fish in each of the four areas both before and after exposure to the OMR stimulus. Control fish show a statistically significant difference between the initial position and the final position, indicating the control larvae respond and align to the OMR animation (Figure 3F, Movie S1). The control larvae change from a random distribution to an enriched distribution on the left side of the plate, with 75% of larvae occupying Areas 3 and 4 (Figure 3F). In contrast, the *mbl^−/−^* fish do not show a difference between their initial position and final position (Figure 3F, Movie S5). Both before and after the OMR animation, the *mbl^−/−^* larvae show a 50% or greater distribution in Area 1. This indicates the *mbl^−/−^* fish are unable to see and process the OMR animation.

We next evaluated both control and *mbl^−/−^* larvae by OMR. We measured the positions of fish before and after 30 s exposure to the OMR animation. We graph the results in two different ways: a population analysis and post-stimulus analysis. The population graph is a bar graph representing the percentages of fish in each of the four areas both before and after exposure to the OMR stimulus. Control fish show a statistically significant difference between the initial position and the final position, indicating the control larvae respond and align to the OMR animation (Figure 3F, Movie S1). The control larvae change from a random distribution to an enriched distribution on the left side of the plate, with 75% of larvae occupying Areas 3 and 4 (Figure 3F). In contrast, the *mbl^−/−^* fish do not show a difference between their initial position and final position (Figure 3F, Movie S5). Both before and after the OMR animation, the *mbl^−/−^* larvae show a 50% or greater distribution in Area 1. This indicates the *mbl^−/−^* fish are unable to see and process the OMR animation.

Our second analysis of the OMR assay is the post-stimulus analysis. This analysis focuses on individual larval response to the OMR. The same area numbers used above (1 to 4) (Figure 2) are converted to ‘position scores’. We take the final position score of the larvae and subtract the starting position score to calculate a change score (Equation (1)). Change scores can therefore take on an integer value from +3 to −3 where a positive score denotes an increase in alignment, zero denotes no change in alignment, and a negative score denotes a decrease in alignment.

The post-stimulus analysis equation is

Position_final_ − Position_initial_ = Post-stimulus analysis score (1)

For the control group, the larvae trend toward positive scores. This indicates the fish align with the direction of animation and relocate from areas 1 and 2 to areas 3 and 4 (Figure 3G). Conversely, the *mbl^−/−^* post-stimulus analysis score is highly skewed around 0, indicating the fish either did not change their position or they left and returned to the same area, in either case, this reflects a non-response to the OMR (Figure 3G).

### 3.3. Candidate Gene Selection: crim1 and rbm24a

**Figure 4:**
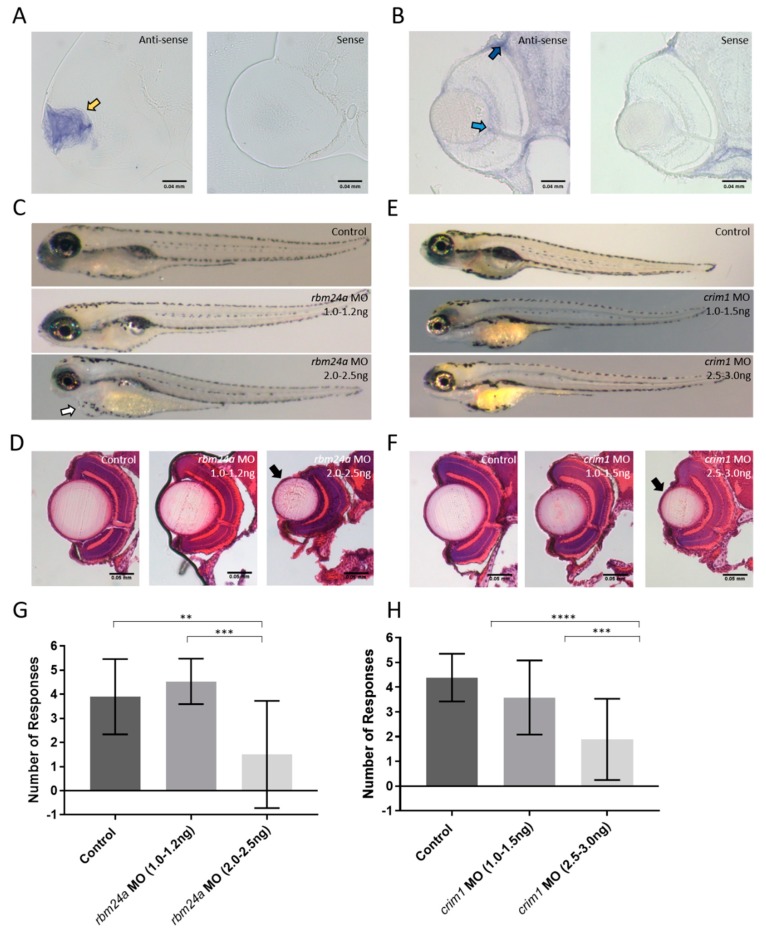
Analysis of candidate genes. Whole mount in situ hybridization on 3 dpf retinal sections for (**A**) *rbm24a*; (**B**) and *crim1*; (**C**) Phenotypes of control, *rbm24a* AUG MO at 1.0–1.2 ng, and *rbm24a* AUG MO at 2.0–2.5 ng; (**D**) H and E staining of control, *rbm24a* AUG MO at 1.0–1.2 ng, and *rbm24a* AUG MO at 2.0–2.5 ng; (**E**) Phenotypes of control, *crim1* AUG MO at 1.0–1.5 ng, and *crim1* AUG MO at 2.5–3.0 ng; Same magnification as (**A**); (**F**) H and E staining of control, *crim1* AUG MO at 1.0–1.5 ng, and *crim1* AUG MO at 2.5–3.0 ng. Same magnification as (**B**); (**G**) VIZN analysis of control (*n* = 19), *rbm24a* AUG MO at 1.0–1.2 ng (*n* = 18), and *rbm24a* AUG MO at 2.0–2.5 ng (*n* = 10) (Mann–Whitney *p*-value ** = 0.0068, *** = 0.0003); (**H**) VIZN analysis of control (*n* = 39), *crim1* AUG MO at 1.0–1.5 ng (*n* = 31), and *crim1* AUG MO at 2.5–3.0 ng (*n* = 18) (Mann–Whitney *p*-value **** < 0.0001). Yellow arrow indicates lens staining. Light blue arrow denotes ganglion cell layer staining. Dark blue arrow indicates choroid staining. Black arrows point to microphthalmic eyes. White arrow indicates cardiac edema. Scale bars in (**A**) and (**B**): 0.04 mm, and in (**D**) and (**F**): 0.05 mm.

We identified two candidate genes to investigate: *rbm24a* and *crim1*. Both of these genes had been previously shown to have general expression in the eye [25,43]. To determine the more precise localization of these candidate genes, we performed whole mount in situ hybridization and sectioned the eyes of the embryos. The expression for *rbm24a* is exclusively in the lens while *crim1* is found in the ganglion cell layer and choroid (Figure 4A,B).

Both *rbm24a* and *crim1* have roles in other organs which hampers the analysis of visual function. To avoid complications from other organ defects, we utilized low-dose morpholino oligonucleotides (MO) to create partial knockdown. These morphants appear phenotypically normal. We ensured MO injections themselves did not contribute to visual defects by injecting a control MO. In our control MO injected larvae, we find normal morphology and retinal lamination (Figure S1A,B). We evaluated these morphants for VIZN and OMR and found the control MO injected embryos did not differ from uninjected control larvae (Figure S1C–F). We next sought to analyze our candidate genes.

### 3.4. Analysis of rbm24a and crim1 Morphant Phenotypes and Response to VIZN

To analyze the function of *rbm24a* and *crim1*, we used translation-blocking mopholinos (MO). We injected a narrow dose range of the *rbm24a* MO: 1.0–1.2 ng and 2.0–2.5 ng. The 1.0–1.2 ng injection appeared phenotypically normal while roughly 80% of the 2.0–2.5 ng displayed microphthalmia with 25% also displaying cardiac edema (Figure 4C). These phenotypes are reminiscent of those observed in knockout mouse mutant models [19]. Retinal sections of morphants were examined by hemotoxylin and eosin (H&E) staining. Sections containing an optic nerve were analyzed except the *rbm24a* 2.0–2.5 ng dose. The control and *rbm24a* 1.0–1.2 ng morphants looked indistinguishable from each other, while the 2.0–2.5 ng dose displayed severe microphthalmia (Figure 4D).

We performed a similar dose range experiment with the *crim1* MO. At the 1.0–1.5 ng dose, the injected embryos appear phenotypically normal. At the higher dose of 2.5–3.0 ng, nearly 80% the morphants show microphthalmia (Figure 4E). Eye sections of the *crim1* morphants were analyzed by H&E staining. The lamination of the 1.0–1.5 ng morphants and control appear normal. However, 2.5–3.0 ng MO injected embryos display micropthlamia (Figure 4F).

Patients affected by microphthalmia and cataracts often retain some visual function. We tested morphant larvae for each candidate gene by VIZN. Both *rbm24a* and *crim1* show a dose-dependent response to VIZN. Both lower dose morphants of *rbm24a* (1.0–1.2 ng) and *crim1* (1.0–1.5 ng) respond the same as the control larvae (Figure 4G,H). Our data indicate that the low dose knockdowns of *rbm24a* and *crim1* retain light perception. However, both *rbm24a* and *crim1* VIZN scores at higher doses (2.0–2.5 ng and 2.5–3.0 ng respectively) are statistically significantly different from both the control and the lower dose counterparts (Figure 4G,H). The activity plots from VIZN also a dose-dependent response to the vision startle (Figures S2 and S3). The lack of response in the VIZN assay by larvae injected with the higher doses of *rbm24a* and *crim1* MO is consistent with the morphological phenotypes observed. Moreover, our data indicate that the low dose *rbm24a* and *crim1* morphants retain light perception. As VIZN only tests for a larva’s response to a stark change in light, we next evaluated the possibility of subtle visual defects in the *rbm24a* and *crim1* low-dose morphants by OMR.

### 3.5. rbm24a Morphants Are Visually Impaired by OMR

**Figure 5:**
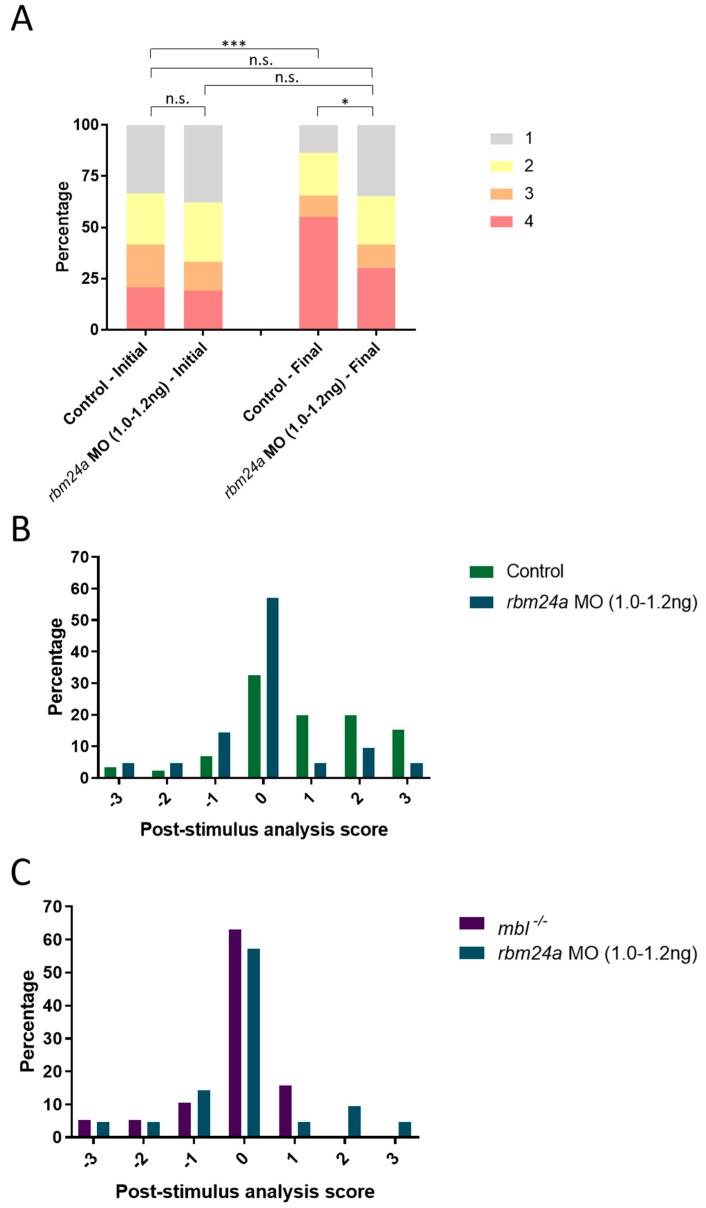
*rbm24a* morphants are visually impaired and unable to respond to OMR. (**A**) OMR analysis of control (*n* = 19), *rbm24a* AUG MO at 1.0–1.2 ng (*n* = 18), and *rbm24a* AUG MO at 2.0–2.5 ng (*n* = 10) larvae plotted as a bar graph which shows the shifts in the population between the initial and final positions (Bowker’s test of symmetry *p*-value *** = 0.0004; Wilcoxon–Mann–Whitney *p*-value * = 0.0094); (**B**) Individual analysis of *rbm24a* morphant compared to wild type; (**C**) individual analysis of *rbm24a* morphant compared to *mbl^−/−^*. In contrast to wild type fish, *rbm24a* morphant fish do not align with the OMR, indicating a visual defect. n.s., not significant.

We performed OMR on control and *rbm24a* 1.0–1.2 ng morphants. We analyzed the data both with the population analysis and post-stimulus analysis. Prior to OMR stimulation both control and *rbm24a* morphants begin randomly distributed between the four areas. At the final position, control larvae show a statistically significant shift from random to highly aligned (Figure 5A). The *rbm24a* morphants’ final positions do not vary significantly from their initial positions. Additionally, the final positions of the control larvae are statistically significantly different from the *rbm24a* morphants’ final positions, further supporting the *rbm24a* morphants are non-responsive to the OMR.

We next analyzed the data by post-stimulus analysis and compared their response to control and *mbl^−/−^* larvae. The change in position for the *rbm24a* morphant larvae was graphed against the control larvae. Unlike the control larvae which trend positively, the *rbm24a* morphants are centered closer to 0 (Figure 5B). We then graphed the *rbm24a* morphants against the *mbl^−/−^* mutants and found both groups display a similar profile in that they are centered around 0 (Figure 5C). This indicates that while the *rbm24a* morphants are visually competent from their VIZN test, they do present a visual defect by OMR.

### 3.6. crim1 Morphants Are Visually Normal by OMR

**Figure 6:**
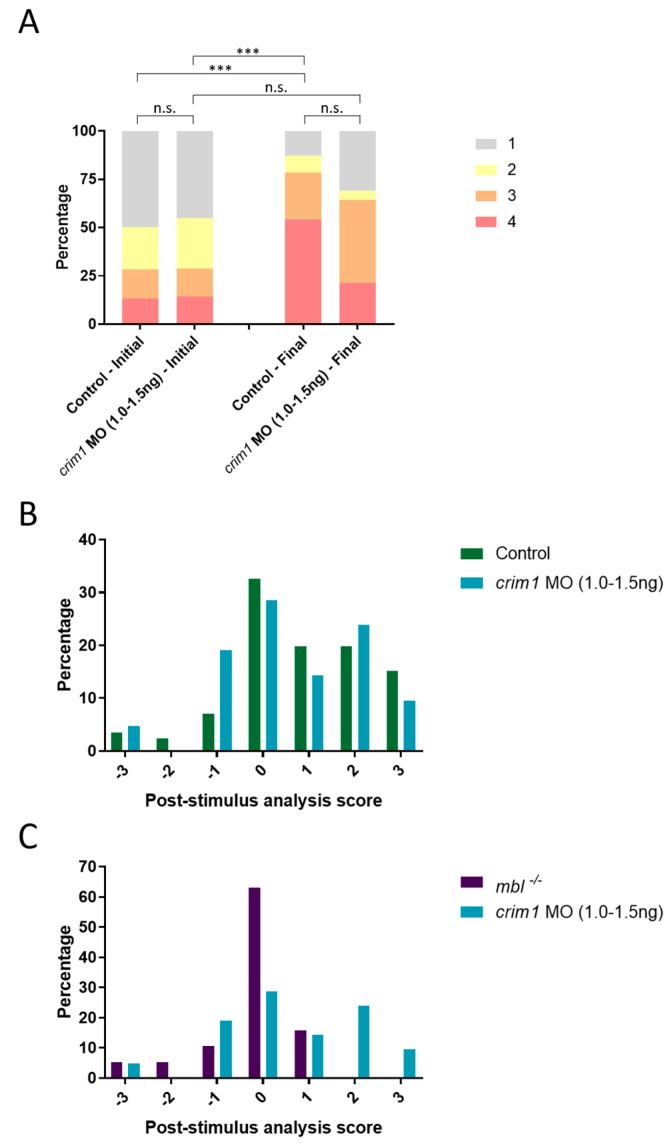
*crim1* morphants respond to OMR. (**A**) OMR analysis of control (*n* = 39) and *crim1* AUG MO at 1.0–1.5 ng (*n* = 31) larvae plotted as a bar graph which shows the shifts in the population between the initial and final positions (Bowker’s test of symmetry, *p*-value *** = 0.0004); (**B**) Individual analysis compared to wild type; (**C**) individual analysis compared to *mbl^−/−^*. Control and *crim1* morphant larvae respond to the OMR. n.s., not significant.

We performed the same OMR assay and analysis on the *crim1* 1.0–1.5 ng morphants. We analyzed the data using both population and individual analysis. Initially, control and *crim1* morphant larvae are randomly distributed. After exposure to the OMR stimulus, the control final positions vary statistically from the control initial positions (Figure 6A). There was no statistically significant difference between the control final positions and *crim1* morphants’ final position, indicating both groups respond to the OMR animation (Figure 6A).

The post-stimulus analysis showed the control and *crim1* morphant larvae both trend in the positive direction (Figure 6B). We also compared the *crim1* morphants to the *mbl^−/−^* mutant larvae. The *mbl^−/−^* mutants cluster near 0 and do not overlap with the positively trending *crim1* morphants (Figure 6C). These data suggest the *crim1* 1.0–1.5 ng morphants are visually comparable to the control larvae. We hypothesized the low-dose injection of *crim1* MO produces a mild cataract phenotype that does not disrupt visual function.

### 3.7. rbm24a and crim1 Morphants Have Congential Eye Defects

**Figure 7:**
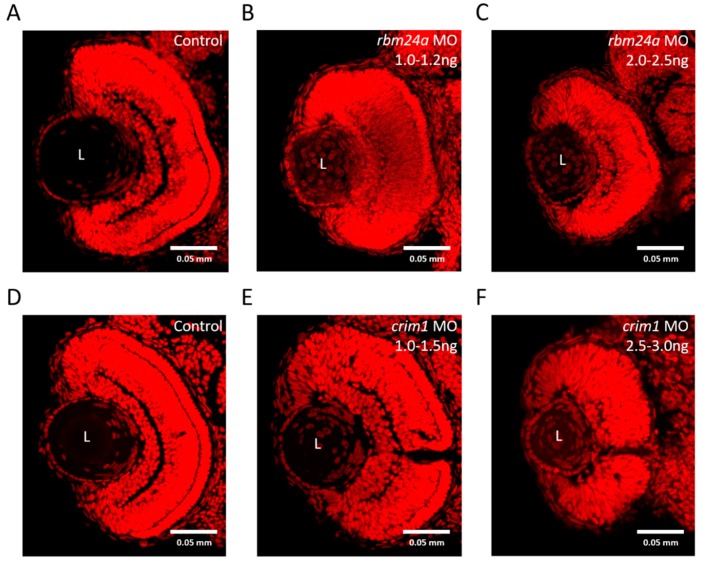
*rbm24a* and *crim1* morphants have congenital eye defects. (**A**) Control; (**B**) *rbm24a* AUG MO 1.0–1.2 ng; (**C**) *rbm24a* AUG MO 2.0–2.5 ng; (**D**) control; (**E**) *crim1* AUG MO 1.0–1.5 ng, and (**F**) *crim1* AUG MO 2.5–3.0 ng. L denotes the lens. Scale: 0.05 mm. TOPRO3-stained nuclei appear red.

We wanted to examine the effect of *rbm24a* and *crim1* knockdown on the development in the early eye. Currently, the only information on potential eye defects from *rbm24a* depletion in an animal model is “eye defects” [23]. We utilized the same dose range of *rbm24a* MO injections described above to investigate lens defects. The differentiated zebrafish lens has very few nuclei [50]. Zebrafish mutants with lens opacity defects often present with an accumulation of nucleated cells in or adjacent to the lens [51,52]. We sectioned 3 dpf zebrafish, stained for nuclei with TO-PRO^TM^-3, and examined the sections via fluorescent confocal microscopy. Control larvae display lenses with very few nuclei in them, as previously reported (Figure 7A). Low-dose knockdown of *rbm24a* at 1.0–1.2 ng yielded nuclei-in-lens cataracts, mild micropthalmia, and some disruption to retinal lamination (Figure 7B). At the higher dose of *rbm24a* knockdown of 2.0–2.5 ng, we observed a similar but more severe phenotype compared to the lower dose and noted nuclei-in-lens cataracts, micropthalmia, and retinal lamination disruption (Figure 7C). From this experiment, we can conclude *rbm24a* knockdown leads to severe micropthalmia, improper retinal lamination, and congenital cataracts. Taken together, we postulate the defects present in the low-dose knockdown are mild enough to maintain light perception and respond to VIZN but limit response to OMR.

For *crim1*, we wanted to confirm our hypothesis that the *crim1* morphants had cataracts akin to the mouse model [37]. We utilized the same dose range of *crim1* MO described above. As previously mentioned, the differentiated zebrafish lens has very few nuclei [50]. Again, control zebrafish display very few cataracts at 3 dpf (Figure 7D). At the low dose of *crim1* 1.0–1.5 ng, larvae have increased nuclei clustered in the medial portion of the lens (Figure 7E). The higher dose of *crim1* MO, 2.5–3.0 ng, leads to a greater increase of nuclei in the lens coupled with microphthalmia and defective lamination (Figure 7F). Analysis of the opacity of the lens leads us to conclude that low-dose *crim1* knockdown induces a mild form of cataracts but is still visually responsive to VIZN and OMR.

## 4. Discussion

Gene discovery for vision disorders will benefit greatly from high-throughput assays that detect blindness as well as visual impairment. Here we demonstrate an adaptation of the optomotor response (OMR) that can be efficiently performed in multi-well plates with minimal cost. Materials for the adapted OMR are simple: a tablet to display the animation, a mount for a cell phone or other video camera, multi-well plates for the larvae, and an investigator to score the positions of the fish. Additionally, the assay is well suited for high-throughput analysis. We combine the VIZN and OMR assays for a comprehensive analysis of vision. For labs that do not have tracking devices, this adapted OMR can be run alone. Taking into account the steps for data collection, performing the OMR requires minimal labor and time. The manual loading of larvae into 48-well plates takes roughly 15 min, then we recommend acclimating fish to their environment for 2 or more hours, before they are assayed for OMR, which takes roughly 1 min. Because multiple plates can be acclimated at the same time, we consider a 48-well plate of fish requires about 20 min to load and perform OMR. By extrapolation, we estimate that an individual could process more than 1000 fish in a single 8 h day. And finally, scoring the OMR videos takes~20 min for each 48-well plate. 

We demonstrated the use of the *mbl^−/−^* zebrafish as a negative control in visual studies. We first determined the eyeless *mbl^−/−^* mutants have an intact swimming circuit before testing with VIZN, for light perception, and OMR, for motion detection [7]. While the eyeless *mbl^−/−^* larvae responded to touch with a normal escape response, they showed little to no response to the flashes of light during VIZN nor to the animated black and white lines in OMR, making them a negative control for visual studies. Other zebrafish genetic mutants with vision defects can be used as a negative control, as long as the animals maintain motility.

Utilizing the VIZN and OMR assays, we investigated two genes related to visual impairment: *rbm24a* and *crim1*. A challenge to this study was that both *rbm24a* and *crim1* have multiple roles in organs outside the eye. Homozygous mutants for both genes are embryonic or postnatal lethal, rendering these homozygotes unsuitable for visual studies. To solve this problem, we utilized low-dose morpholino knockdown to assay the visual function while maintaining viable larvae [45]. We showed the ability to monitor visual function in both *rbm24a* and *crim1* morphants using this approach.

The gene *rbm24a* encodes an RNA binding protein that plays key roles in development by binding to and regulating the expression of target mRNAs [20,21,22,23]. Knockdown of *rbm24a* results in microphthalmia and retinal lamination defects along with congenital cataracts which worsen at increased MO doses. We used low-dose knockdown to generate phenotypically normal zebrafish to simulate subtle microphthalmia. These larvae are responsive in the VIZN assay. However, when we performed OMR on these low dose *rbm24a* morphants, the larvae responded similar to the *mbl^−/−^* mutants. This suggests that while *rbm24a* morphants resemble their control counterparts morphologically and in the VIZN assay, they have a vision impairment. The extent to which this is caused by decreased visual acuity due to the slightly smaller eye or a defect in visual processing remains to be determined.

It is possible that the microphthalmia displayed by *rbm24a* morphants is due to an increase in cell death. RNA binding proteins are known to have roles regulating apoptosis through either increasing or decreasing the amount of apoptotic activity [53,54,55]. Previous studies have demonstrated increased apoptosis in the eye can lead to lamination defects similar to those we observed in *rbm24a* morphants [56,57]. Additionally, while no animal model has described the eye defect associated with *rbm24a* depletion, we find evidence that reduced *rbm24a* expression could lead to cataract formation. Previous research has implicated several RNA binding proteins in the development of cataracts [23,58,59,60,61].

The zebrafish *crim1* encodes a transmembrane protein which interacts with members of growth factor families which include TGFβs, BMPs, and VEGFs [37,41]. Knockdown of *crim1* leads to nuclei-in-lens cataracts. Increased knockdown, using higher MO dose, leads to more nuclei in the lens along with microphthalmia. We again used a low dose knockdown strategy which provided phenotypically normal larvae. These larvae were visually responsive to VIZN. We next performed OMR on these low dose morphants. Despite the presence of cataracts, the low dose *crim1* morphants responded almost as well as control larvae. These data suggest that while cataracts can impair vision, cataracts at the less severe end of the spectrum can allow for near normal visual acuity.

Our adaptation and validation of the OMR assay provides a cheap and easy-to-use visual assay. We demonstrate an approach using a simple set up with a cell phone camera and tablet. The assay is adaptable. For example, we defined four areas for scoring in a 48-well plate. The type of multi-well plate and the areas used for scoring alignment can be modified to fit experimental needs. Additionally, it would be ideal to automate the OMR. One drawback of the current iteration of the OMR is that the moving black and white lines in the video are not well-suited for tracking software. An infrared camera could be substituted in place of a cell phone or normal video camera. The infrared camera can detect the larvae and track them without interference from the OMR animation. This would allow the use of motion-tracking software to automatically track fish and allow the user to examine the paths of individual fish over entire time course, providing additional behavior data.

In early iterations of the OMR, we tried several variations to align the fish prior to the start of the animation. We explored a ‘race track’ in which small groups of fish were positioned on one side of a plastic gate. We would then start the OMR animation and pull the gate. Unfortunately, the action of pulling the gate induced a startle response in the fish which disrupted their initial orientation. In another approach, we sought to expose the fish to the OMR animation in one direction, moving right to left, and then switch the direction of the animation to run left to right. The intent was that visually responsive fish would align to the left and then align to the right, providing a robust analysis of motion detection. However, when the lines would change directions, the fish would startle and become randomly oriented. Future development could explore a mechanism to align the fish initially without inducing a startle response.

Additional uses for the OMR assay include genetic screens. CRISPR-mediated mutagenesis can generate many different alleles and if overall morphology is normal, characterizing multiple alleles can be time consuming. The OMR response of individual larvae can be followed by a genotype assessment to accelerate allele characterization. In addition, the multi-well plate OMR assay can readily be used to test chemical modifiers in screens to identify reagents that enhance or reduce visual function.

## Supplementary Materials

Supplementary materials can be found at https://zenodo.org/record/2636986#.XLbpSmiYOpq. Figure S1: Standard Control morphology, VIZN, and OMR; Figure S2: VIZN activity plots for *rbm24a* morphants; Figure S3: VIZN activity plots for *crim1* morphants; Video S1: Control response to OMR 12 well plate; Video S2: *mbl* homozygous mutant response to OMR 12 well plate; Video S3: Control touch response; Video S4: *mbl* homozygous mutant touch response; Video S5: *mbl* het or homozygous sibling touch response.
